# The effect of gabapentin and pregabalin on intestinal incision wound healing in rabbits

**DOI:** 10.14202/vetworld.2015.279-283

**Published:** 2015-03-07

**Authors:** M. Korkmaz, T. B. Saritas, A. Sevimli, Z. K. Saritas, B. Elitok

**Affiliations:** 1 Department of Surgery, Faculty of Veterinary Medicine, Afyon Kocatepe University, 03200, Afyonkarahisar, Turkey; 2 Department of Anaesthesiology and Reanimation, Meram Medical School, Necmettin Erbakan University, 42080, Konya, Turkey; 3 Department of Pathology, Faculty of Veterinary Medicine, Afyon Kocatepe University, 03200, Afyonkarahisar, Turkey; 4 Department of Internal Medicine, Faculty of Veterinary Medicine, Afyon Kocatepe University, 03200, Afyonkarahisar, Turkey

**Keywords:** gabapentin, intestinal incisional wound, pregabalin, rabbit

## Abstract

**Aim:**

To evaluate the macroscopic and histologic effects of pregabalin (PG) gabapentin (GB) on longitudinal intestinal wound healing in New Zealand rabbits.

**Materials and Methods:**

The animals were divided into three groups randomly; the control group (n=6), PG group (n=6) and GB group (n=6). All animals were premedicated with xylazine HCI, 5 mg/kg i.m. and general anaesthesia was performed by ketamine HCI 50 mg/kg i.m injection. A 4 cm incision in the caecum through median laparotomy was achieved under aseptic surgery. Intestinal wound was closed with double-sutured. All animals were received parenteral antibiotic treatment for 5 days. PG and GB groups were treated by PG (30 mg/kg, oral, daily) and GB (30 mg/kg, oral, daily) for 10 days respectively. Control group did not receive any treatment. The animals were euthanized on day 10 and the caecum was examined by laparotomy. Adhesion formation was observed, and tissue samples were taken from suture lines for histologic examination. Cellular infiltration (polymorphonuclear white blood cells and mononuclear cells), accumulation of connective tissue, vascularization and extent of necrosis were evaluated and scored separately for each of mucosal, submucosal, muscular and serosal layers of caecum.

**Results:**

Adhesions were more severe in the GB group compared to other groups. No statistically significant differences were detected among the three groups about the wound healing.

**Conclusion:**

It was suggested that the use of gabapentinoids had no significant effect on wound healing in patients undergoing gastrointestinal surgery and further studies with treatment periods longer than 10 days are needed.

## Introduction

Wound healing is a complex process that the ­tissue repairs itself [1]. The wound healing process is similar in various tissues; however, gastrointestinal system has some distinguishing features, such as tension time that develops much earlier in a gut wound than in the skin [2]. Another point is the synthesizing of collagen by smooth muscle cells in intestinal wounds [3]. Intestinal wound healing essentially includes the stages of inflammation, proliferation-fibroplasia and maturation. Inflammation starts with vasodilation, secretion of vasoactive substances and increase of vascular permeability and neutrophil infiltration within 3 h, following vasoconstriction of the wound margins. Then macrophages and fibroblasts migrate to the wound area. Macrophages regulate inflammation by releasing cytokines [4,5]. Also numerous systemic and local factors play a main role in the healing of intestinal wounds [6]. Important factors in the extracellular matrix are collagen fibers, fibroblasts and immune cells that regulate wound strength in the early postoperative healing process [6,7].

Post-operative pain is one of the most common concerns in surgery. According to some reports, pain treatment is inadequate in nearly half of the patients [8]. A multimodal approach to analgesia is generally recommended for considering of pain that arises through various mechanisms. Opioids, local anaesthetic agents, nonsteroidal anti-inflammatory drugs (NSAIDs), paracetamol and gabapentinoids are only a few of the drugs used in pain management [9,10]. Gabapentinoids are within the spectrum of anticonvulsant agents. Several studies have mentioned their role in the treatment of acute pain [11-13]. Pregabalin (PG) and gabapentin (GB) are the two main representatives of the gabapentinoids group. They are both commonly used as a part of multimodal analgesia in the postoperative period [14,15]. The possible effects of gabapentinoids on intestinal incisional wound healing have not been studied yet, however thus this study was focused on this issue.

## Materials and Methods

### Ethical approval

A total of 18 adult male New Zealand rabbits weighing between 3500 and 4000 g were used in the study after the approval of the Local Ethical Committee for Experimental Animals, Afyon Kocatepe University (Authorization Number:198 and Date: 14.06.2012). All experimental manipulations were performed, and post-operative care administered in accordance with the National Institutes of Health Guide for the Care and Use of Laboratory Animals.

### Experimental procedure

The animals were randomly allocated into three groups as a control group (n=6), PG group (n=6) and GB (n=6). General anaesthesia was performed by i.m. administration of ketamine hydrochloride 50 mg/kg (Alfamine 10%, Egevet, Turkey) followed by xylazine hydrochloride premedication, 5 mg/kg (Alfazine 2%, Egevet, Turkey) i.m. application.

Animal was placed on the operating table in dorsal recumbency. The abdominal area was prepared for aseptic surgery. A 3-4 cm length incision was performed in caecum and then closed with 4/0 prolene in a double suture manners. Then, abdominal wall was closed by routine surgical technique; the wound was provided appropriate care to end of the study. Antibiotic treatment was also applied to all animals for 5 consecutive days.

PG and GB groups were treated by PG (30 mg/kg, oral, daily) (Lyrica, Pfizer, Turkey) and GB (30 mg/kg, oral, daily) (Neruda, Sanovel, Turkey) for 10 days respectively. Control group did not receive any treatment.

All rabbits were euthanized by administration of intravenously given 150 mg thiopental (Pental, 0.5 g vials, I.E. Ulugay, Turkey) and a median re-laparotomy was performed at the end of day 10. Intraperitoneal adhesions were scored according to the Evans scoring system [16]. Accordingly, the extent of adhesions was evaluated as follows; 0: no adhesion, 1: firmly and avascular adhesions separating spontaneously, 2: firm and limited vascular adhesions separated by traction, 3: dense adhesions separating by sharp dissection.

### Histological evaluation

Tissue samples were collected from the sutured intestinal area for histologic evaluation. Cecal tissue samples were fixed in a 10% formaldehyde solution. They were treated by the routine preparation methods and embedded in paraffin; 4-5 µm thick sections were stained with haematoxylin-eosin and examined under light microscopy.

In histopathology, cecal lesions were assessed as follows: Cellular infiltration (polymorphonuclear white blood cells [WBC] and mononuclear cells), accumulation of connective tissue, vascular formation, presence and extent of necrosis were evaluated and scored separately for each of mucosal, submucosal, muscular and serosal layers of caecum. Scoring criteria was follows as: 0%: absent (0), if <25%: mild intensity and extension (+1), if 25-50%: moderate intensity and extension (+2), if >50%: severe (+3).

### Statistical analysis

Data were analysed with the SPSS 16.0 (SPSS Inc, for Windows) software package. Comparison of wound healing and adhesion scores between groups was assessed by the Kruskal–Wallis test. Descriptive results are expressed as mean ± standard deviation. For all comparative tests, p<0.05 was considered significant.

## Results

No postsurgical complication such as death or surgical wound infection was observed in the study.

**Figure-1:**
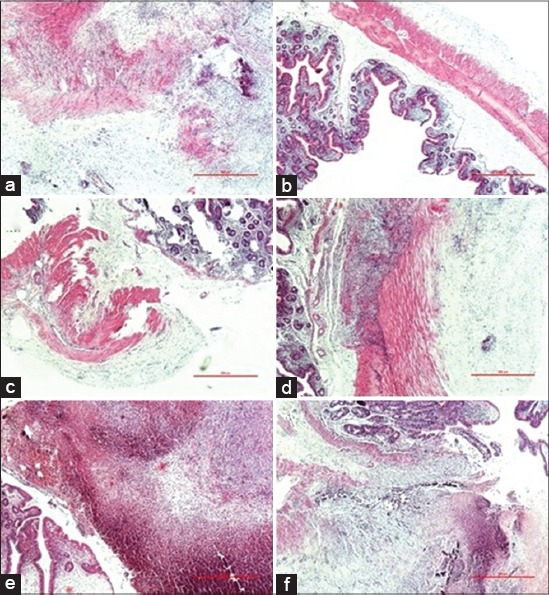
(a) Animal C1, thickening of the muscular and submucosal layer of caecum, serosal vascular proliferation. (b) C3, slight infiltration in the mucosa and submucosa, normal serosa. (c) PG2, slight mucosal infiltration, slight infiltration and vascular proliferation in the serosa. (e) GP3, necrosis and thickening of submucosal, muscular and serosal layer of caecum. (f) GP5, mucosal, submucosal, muscular and serosal layer showing diffuse thickening and vascular proliferation. Haematoxylin-eosin staining.

Histological evaluation of tissue samples obtained from control group showed severe and extensive connective tissue proliferation mainly consisting of neutrophil WBC and slightly mononuclear cell infiltration, an increase of connective tissue formation, presence of vascular proliferation and focal necrotic areas (Table-1, Figure-1a and b).

**Table-1:** The scoring of histologic data obtained from mucosal, submucosal, muscular and serosal layer of caecum in control (C) (n=6), PG (n=6) and GB (n=6) groups

Groups	Mucosal layer	Submucosal layer	Muscular layer	Serosal layer
C	2.33±0.81	2.66±0.81	2.66±0.81	2.83±0.40
PG	2.33±0.51	2.66±0.51	2.83±0.40	2.83±0.40
GB	2.44±0.61	2.83±0.40	3±0.00	3±0.00

PG=Pregabalin, GB=Gabapentin, C=Control, Extent of cellular infiltration (polymorphonuclear WBC and mononuclear cells), accumulation of connective tissue, vascular proliferation, and presence and extent of necrosis were graded according to a score system; 0%: absent (0), if <25%: mild intensity and extension (+1), if 25-50%: moderate intensity and extension (+2), if >50%: severe (+3). No significant difference was detected between groups (p>0.05), WBC=White blood cells

In the PR group, lesions such as connective tissue proliferation (consisting mainly of neutrophil WBC and mononuclear cells), accumulation of connective tissue, presence of vascular proliferation and necrosis of mainly moderate severity were evident in mucosal layer, while the lesions in the submucosal, muscular and serosal layer were mostly severe (Table-1, Figure-1c and d).

GB group animals displayed severe and extensive neutrophilic (with slightly lesser mononuclear cell) infiltrates, accumulation of connective tissue formation, vascular proliferation and necrosis in mucosal, muscular and the serosal layer of caecum (Table-1, Figure-1e and f). Statistical evaluation did not reveal any significant difference between the groups about histologic features (p>0.05).

Adhesion was seen between the incision line and the omentum or other surrounding structures in one animal in both control and PG groups and three cases in GP group. Adhesion scores were 0.33±0.8, 0.33±0.8, 1±1.1 in control, PG and GB groups respectively. Moreover, adhesion scores were not statistically significant among the groups. Adhesions along the suture line were more frequent in GP group.

## Discussion

Several systemic and local factors such as blood transfusions [17], ischemia, infection, diabetes mellitus, hypovolemia and anaemia may affect ­intestinal wound healing negatively [18,19]. On the other hand, it is well known that NSAIDs may have a positive effect on anastomosis healing by reducing prostaglandin synthesis whereas corticosteroids delay postoperative healing of anastomotic wounds by suppressing macrophage function and inflammation [4,20]. The effect of drugs which are used post-operatively, including intravenous, inhaled and local anaesthesia agents [21,22], such as opioids, NSAIDs, muscle relaxants, corticosteroids, antiemetics or gabapentinoids is also highly relevant to wound healing. Some of the local anaesthetics [22], opioids [23], corticosteroids [20] and inhaled anaesthesia agents have been studied in this regard [24]. The influence of gabapentinoids on intestinal incisional wound healing is still unknown, notwithstanding the fact of their widespread use for perioperative and postoperative pain. This is the first study evaluating the effect of PB and GB of intestinal incisional healing. Accordingly, it was determined that the results of intestinal incisional healing in both PG and GB groups as compared to the control group did not show any significant difference.

The most common complications of gastrointestinal operations are anastomotic leakage [25,26] and postsurgical peritoneal adhesions [27,28]. High morbidity and mortality are most frequently resulted by anastomotic leakages. Peritoneal adhesions can cause especially the morbidity including intestinal ­obstruction, infertility and the chronic abdominopelvic pain and are associated with multiple surgical complications [28]. In this study intestinal leakage and another any surgical complications or infections were not observed in all groups. Similar to other reports [27,28] peritoneal adhesions between the incision line and the omentum or other surrounding tissues along the intestinal suture line were seen in one animal in both control and PG groups and three cases in GP group.

Inhalation anaesthesia agents are a major class of drugs used in surgery. Their effects on wound healing and inflammation were studied by several authors. Yang *et al*. [29] reported that such agents, especially isoflurane, triggered tissue injury by over activating inositol trisphosphate receptors, which leads to abnormal calcium secretion from the endoplasmic reticulum. Isoflurane, halothane and enflurane suppress the inflammatory response by reducing proinflammatory cytokines [30]. Lee *et al*. [21] studied the effect of sevoflurane exposure for longer than 4 h on the wound healing stage. They showed a reduction in superficial expression of transforming growth-factor beta-β_1_ and basic fibroblast growth factor and a delay in the shrinking of the wound dimensions. These investigators established that an 8-h exposure to sevoflurane delayed wound healing, compared to oxygen exposure [21]. These reports consideration that wound healing may be delayed proportionally to exposure time in patients undergoing general anaesthesia by inhalation, led us to avoid inhalation anaesthesia in rabbits. Therefore, we used xylazine and ketamine in order to avoid some possible negative effects on intestinal incisional wound healing in our study.

PG and GB have been used in the treatment of postsurgical pain, neuropathic pain, epilepsy, spasticity and anxiety. In recent years, PG and GB have been used commonly for acute postsurgical pain treatment [14]. Although the analgesic effect of gabapentinoids is still unclear, it is believed that they show the inhibitory effect via the inhibition of α (2)/δ subunit of voltage-dependent calcium channels. GB also inhibits glutamate release, increases the activity of voltage-gated N-methyl-D-aspartate receptors, and inhibits the activity of voltage-gated potassium channels [31]. PG decreases glutamate, noradrenaline and substance P via inhibiting the influx of calcium [32]. The mechanism of the anti-inflammatory effect, however, is not yet elucidated. Substance P may cause degranulation of mast cells and induce chemotaxis of neutrophils and lymphocytes [33]. The inhibition of substance P by gabapentinoids may be the one of the mechanisms of the anti-inflammatory effect. It has been suggested that gabapentonoids may negatively affect the molecular mechanisms of wound healing. Moreover, Saritas *et al*. [24] reported that GB was delayed skin wound healing prominently during first 10 days but PG showed more prominent negative effect on skin wound healing between day 10 and 21. Nevertheless in this study, it has been suggested that PG or GB did not have any negative effect on intestinal incisional wound healing, since no significant histopathologic difference detected on 10^th^ day was evident in groups.

## Conclusion

This study addressed the effect of 10 consecutive days of administration of PG or GB on wound healing of a longitudinal intestinal incision. It is suggested that either PG or GB shows a negative effect on intestinal incisional wound healing. However, it is also recommended that further studies with treatment periods longer than 10 days are needed to clarify the harmless effect of PG or GB.

## Authors’ Contributions

MK carried out the study, drafted and revised the manuscript. TB Saritas helped to design the study and participated in scientific discussion. ZKS helped to design the study and experimental examinations. AS analysed all the histopathological data. BE helped to analyze the data. All the authors read and approved the final manuscript.
